# Innate sensing of viruses by pattern recognition receptors in birds

**DOI:** 10.1186/1297-9716-44-82

**Published:** 2013-09-09

**Authors:** Shun Chen, Anchun Cheng, Mingshu Wang

**Affiliations:** 1 Institute of Preventive Veterinary Medicine, Sichuan Agricultural University, Chengdu, Sichuan 611130, China; 2 Avian Disease Research Center, College of Veterinary Medicine, Sichuan Agricultural University, 46 Xinkang Road, Ya’an, Sichuan 625014, China; 3 Key Laboratory of Animal Disease and Human Health of Sichuan Province, Sichuan Agricultural University, Chengdu, Sichuan 611130, China

## Abstract

Similar to mammals, several viral-sensing pattern recognition receptors (PRR) have been identified in birds including Toll-like receptors (TLR) and retinoic acid-inducible gene I (RIG-I)-like receptors (RLR). Avian TLR are slightly different from their mammalian counterparts, including the pseudogene TLR8, the absence of TLR9, and the presence of TLR1La, TLR1Lb, TLR15, and TLR21. Avian TLR3 and TLR7 are involved in RNA virus recognition, especially highly pathogenic avian influenza virus (HPAIV), while TLR15 and TLR21 are potential sensors that recognize both RNA viruses and bacteria. However, the agonist of TLR15 is still unknown. Interestingly, chickens, unlike ducks, geese and finches, lack RIG-I, however they do express melanoma differentiation-associated gene 5 (MDA5) which functionally compensates for the absence of RIG-I. Duck RIG-I is the cytosolic recognition element for HPAIV recognition, while chicken cells sense HPAIV through MDA5. However, the contributions of MDA5 and RIG-I to IFN-β induction upon HPAIV infection is different, and this may contribute to the chicken’s susceptibility to highly pathogenic influenza. It is noteworthy that the interactions between avian DNA viruses and PRR have not yet been reported. Furthermore, the role for avian Nod-like receptors (NLR) in viral immunity is largely unknown. In this review, recent advances in the field of viral recognition by different types of PRR in birds are summarized. In particular, the tissue and cellular distribution of avian PRR, the recognition and activation of PRR by viruses, and the subsequent expression of innate antiviral genes such as type I IFN and proinflammatory cytokines are discussed.

## Table of contents

1. Introduction

2. Toll-like receptors

2.1 Avian TLR 1, 2, 4, and 5

2.1 Avian TLR 15

2.1 Avian nucleic acid-sensing TLR

2.2 Avian TLR3

2.3 Chicken TLR3

2.3 Other avian TLR3

2.2 Avian TLR7

2.3 Chicken TLR7

2.3 Duck TLR7

2.3 Goose TLR7

2.2 Mammalian TLR9 and avian TLR21

3. Rig-I-like receptors for cytosolic sensing

3.1 RIG-I

3.1.1 Duck RIG-I

3.1.1 Goose RIG-I

3.1 MDA5

3.1.1 Chicken MDA5

3.1 Chicken LGP2

4. Nod-like receptors

4.1 NLRC5

5. Conclusions

6. Competing interests

7. Author’s contributions

8. Acknowledgements

9. References

## 1. Introduction

Vertebrates sense viral pathogen-associated molecular patterns (PAMP) through different types of pattern recognition receptors (PRR). The activation of PRR by PAMP triggers the activation of transcription factors and the expression of innate antiviral genes such as interferon, which is central to host antiviral defences. Mammalian and avian lineages diverged approximately 300 million years ago, and therefore their DNA share many evolutionarily conserved regions including many PRR, however the avian genome contains some distinct genes (Table 1) [1]. Studies indicate that avian immune systems differ from their mammalian counterparts [2]. Birds lack functional eosinophils, although avian heterophils functionally replace mammalian neutrophils. Moreover, birds lack lymph nodes, but have some avian-specific primary lymphoid organs such as the bursa of Fabricius, which is the site of hematopoiesis and necessary for B cell development. Also, harderian glands in birds play an important role in adaptive immune responses. Birds and mammals use partly similar and partly distinct molecules for the same immunological antiviral mechanisms including TLR [3,4], defensins [5], cytokines [6], chemokines [6], antibodies and others [7].

**Table 1:** Comparison of the PRR between human and birds

**Pattern recognition**	**Human**	**Origin of ligand**	**Chicken**	**Duck/Goose**	**Origin of ligand**
**receptor (PRR)**					
**Membrane-bound PRR (TLR)**					
**On plasma membrane**	TLR1/6/10	Bacteria	TLR1La TLR1Lb	Not reported	Unknown; bacteria
	TLR2	Bacteria; Fungus; Parasites; Virus	TLR2a TLR2b	Present in duck; Not reported in goose	Unknown; Bacteria
	TLR4	Bacteria; Fungus; Parasites; Virus	Present	Present	Unknown
	TLR5	Bacteria	Present	Not reported in duck; Present in goose	Bacteria
	TLR11	Bacteria; Parasites	Absent	Not reported	Unknown
	Absent		TLR15	Not reported in duck; Present in goose	Bacteria; Virus
**In intracellular vesicles**	TLR3	Virus	Present	Present in duck; Not reported in goose	Virus
	TLR7	Virus	Present	Present	Virus
	TLR8	Virus	Pseudogene		
	TLR9	Virus; Bacteria; Parasites	Absent		
	Absent		TLR21	Not reported	Unknown
**Cytoplasmic PRR**					
**RLR**					
	RIG-I	Virus	Absent	Present	Virus
	MDA5	Virus	Present	Present	Virus
	LGP2	Virus	Present	Present	Virus
**NLR**					
**NOD**	NOD1	Bacteria	Present	Not reported	Unknown
	NOD2	Bacteria	Absent	Not reported	Unknown

The innate immune system acts as the first line of defense against invading viruses and plays an important role in the subsequent activation of antiviral responses. The interaction between the host innate immune mechanism and invading viruses is complicated. The earliest host response to a microbial invader is the nonspecific recognition of viral components by PRR [8]. Many innate immune cells including dendritic cells, macrophages, lymphocytes and non-immune cells, such as endothelial cells, mucosal epithelial cells and fibroblasts contain PRR [8]. Some PRR are distributed in the cytosol, including some types of Toll-like receptors (TLR), retinoic acid-inducible gene I (RIG-I)-like receptors (RLR), and nucleotide-binding oligomerization domain (NOD)-like receptors (NLR), which play important roles in the cytosolic recognition of viral double-strand RNA (dsRNA), single-strand RNA (ssRNA) and DNA (Table 1). TLR are well-known membrane-bound PRR which recognize pathogen-associated lipids, proteins, and nucleic acids. TLR and RLR are important for the production of type I interferons (IFN) and various cytokines, whereas NLR play a major role in the regulation of interleukin-1β (IL-1β) [9].

## 2. Toll-like receptors

TLR are type I transmembrane proteins consisting of an extracellular N-terminal domain containing leucine-rich repeats (LRR) that mediate the detection of PAMP, transmembrane domains, and intracellular Toll-interleukin 1 (IL-1) receptor (TIR) domains that are involved in downstream signal transduction [10]. The recognition of PAMP by TLR triggers the activation of transcription factors and the expression of innate antiviral genes such as type I IFN and proinflammatory cytokines [10].

TLR are among the best-characterized PRR and mediate the recognition of invading pathogens both outside the cell and in intracellular endosomes and lysosomes. Different TLR recognize the different molecular patterns of microorganisms and self-components [11]. Currently, 10 human TLR (huTLR), 13 murine TLR, and 10 chicken (Gallus gallus) TLR (chTLR) (Table 1) have been characterized including chTLR1La and chTLR1Lb [12], chTLR2a and chTLR2b [13], chTLR3 [14], chTLR4 [15], chTLR5 [16], chTLR7 [17], chTLR15 [18], and chTLR21 [19]. However, many waterfowl TLR have yet to be identified. So far, 4 duck (Anas platyrhynchos) TLR (duTLR) including duTLR2 [20], duTLR3 [21], duTLR4 [22], duTLR7 [23], and 4 goose (Anser anser) TLR (goTLR) including goTLR4 [24], goTLR5 [25], goTLR7 [26], and goTLR15 [GenBank accession No. JQ014619.1] have been reported. Avian TLR are slightly different from other vertebrates in a number of ways, including the presence of the pseudogene TLR8, the absence of TLR9, and the presence of chTLR1La, chTLR1Lb, chTLR15, and chTLR21 [12].

### 2.1 Avian TLR 1, 2, 4, and 5

Five avian TLR (chTLR2, 4, 5 and 7) are orthologous to other mammalian genes including duplicated genes chTLR2a and chTLR2b [12], while chTLR1La and chTLR1Lb are unique to birds. Avian TLR5 genes are polymorphic, an attribute that may be associated with the resistance or susceptibility of birds to infectious diseases [27]. It is not believed that TLR1 and TLR5 are involved in the host antiviral immune response.

In mammals, TLR2 and TLR4 are present on the plasma membrane of cells and recognize viral components including envelope proteins and surface hemagglutinin, such as hemagglutinin protein of the varicella virus, herpes simplex virus-1, human cytomegalovirus, and mouse mammary tumor virus [28]. However, the mechanism of recognition of viral pathogens by avian TLR2 or TLR4 remains to be elucidated.

### 2.2 Avian TLR 15

TLR15 is considered as an avian-specific TLR [12,29]. To date, TLR15 has been identified in the chicken [18], turkey [30], Japanese quail [GenBank accession No. HM773176.1], goose [GenBank accession No. JQ014619.1] and zebra finch (Taeniopygia guttata) genomes. ChTLR15 is expressed in the spleen, bursa, and bone marrow of healthy chickens [18], while turkey TLR15 is constitutively expressed in various tissues including the heart, liver, intestine, bursa, bone marrow, muscle and spleen [30].

The expression of chTLR15 is upregulated by heat-killed *S. enterica* serovar Typhimurium in chicken embryonic fibroblasts (CEF), indicating that chTLR15 may play a role in the avian immune response against bacterial infections [18]. It was subsequently demonstrated that chicken IL-1β, not IFN-α, can be significantly upregulated by B- and C-type oligodeoxynucleotides containing unmethylated CpG motifs (CpG-ODNs) through chicken TLR15 via myeloid differentiation primary response gene (88) (MYD88)-dependent pathway in a chicken macrophage (HD11) cell line [31]. Moreover, there may be cross talk between TLR2 and TLR15 leading to a reduction in IL-1β levels [31]. Interestingly, avian TLR15 is also involved in the host antiviral immune response. The expression of chTLR15 in the spleens of chickens infected with Marek’s disease virus (MDV) is significantly downregulated at 28 days post infection (dpi), while chTLR3 and chTLR15 were upregulated in the bursa at 7 and 4 dpi, respectively [32]. Another study showed that the expression of chTLR15 in the spleen is upregulated during the cytolytic phase of MDV infection (5 dpi) [33]. These studies provide evidence for the critical role of chTLR15 in controlling MDV infection in the spleen and bursa during the early phases of infection. Collectively, like mammalian TLR4 or TLR9, avian TLR15 is a potential sensor for the recognition of invading viruses and bacteria. However, the agonist of TLR15 is still unknown, and the subcellular location of avian TLR15 remains to be characterized.

### 2.3 Avian nucleic acid-sensing TLR

**Figure 1:**
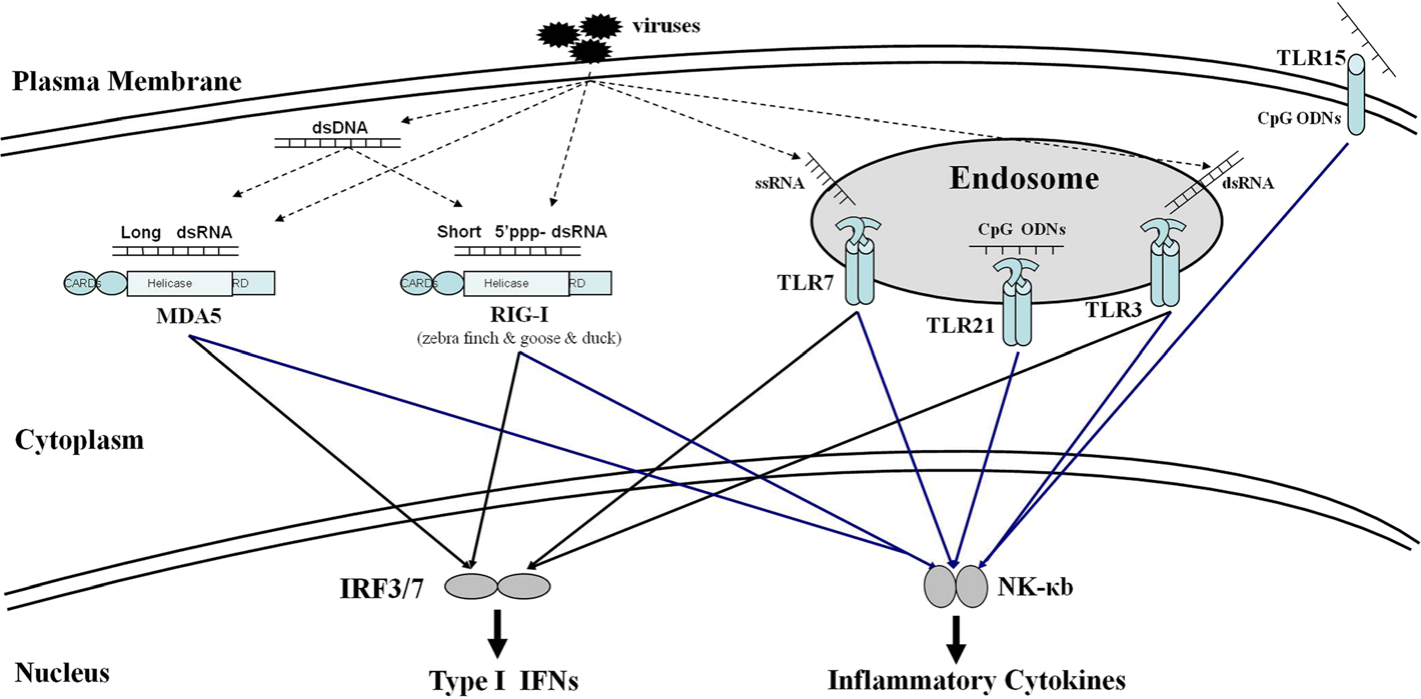
**Model of innate immune recognition of virus in birds.** The plasma membrane receptor of TLR15 recognizes CpG-ODN derived from viruses and bacteria. Viral recognition relies on intracellular vesicles of PRR, whose ligands are dsRNA derived from viruses or virus-infected cells (TLR3), ssRNA derived from RNA viruses (TLR7), CpG-ODN (TLR21), short 5′ppp dsRNA (RIG-I), and long dsRNA (MDA5). TLR3, TLR7 and TLR21 localize mainly in the ER in the steady state and traffic to the endosome, where they engage with their ligands. The recognition triggers the downstream signal transduction to activate NF-κв or IRF3/7, finally induces interferon and inflammatory cytokine production.

Among mammalian TLR, TLR3 and TLR7/8/9 are involved in viral nucleic acid recognition, playing major roles in coordinating antiviral immune responses (Figure 1) [28].

#### 2.3.1 Avian TLR3

TLR3 plays an important role in defending against viral invasion by upregulating the expression of antiviral type I IFN, which has been demonstrated in TLR3-deficient mice [34]. TLR3 recognizes dsRNA formed during viral genome replication or transcription, and is localized exclusively in intracellular vesicles such as the endosome and endoplasmic reticulum (ER) where viruses undergo uncoating during infection (Figure 1) [10].

##### 2.3.1.1 Chicken TLR3

ChTLR3 has approximately 48% amino acid identity with huTLR3 [35], and displays a broad tissue tropism, yet is only moderately expressed in bone marrow, skin, and muscle tissues, similar to the expression profile of mammalian TLR3 [36]. One study indicated that chTLR2, 3, 4 and 21 are present in chicken CD4+ T cells, and chTLR2 and chTLR3 transcripts were discovered in abundance [36]. Therefore, it has been suggested that avian CD4+ T cells may have a potential role in defending against viral pathogens. Moreover, chTLR3 is readily detected in CEF, chicken kidney cells (CKC), chicken B cell-like cell line (DT40) and the HD11 cell line [35]. As it does in mammals, chTLR3 recognizes dsRNA and the agonist poly(I:C), then rapidly induces type I IFN expression (Table 2) [37,38]. The expression of type I IFN and TLR3 in chicken leucocytes is increased after stimulation with poly(I:C), but not poly(C) [37]. Moreover, both IFN-α and IFN-β can upregulate TLR3 expression in a dose-dependent manner both in leucocytes and DF-1 (chicken fibroblast cell line), but not in HD11 cultures [37]. Furthermore, poly(I:C)-induced IFN-β expression is somewhat dependent on chTLR3, which was demonstrated using siRNA gene silencing of chTLR3 [37]. This suggests the presence of alternative pathways to produce IFN-β in chickens, which has also been reported in mammals [34,39]. These studies indicate that chTLR3 is constitutively expressed in all chicken tissues and several kinds of chicken cell lines, and may contribute to the induction of the innate immune response against viruses both in vivo and in vitro.

**Table 2:** The viral sensing PRR in birds

**Viral sensing**		**TLR3**	**TLR7**	**TLR15**	**TLR21**	**RIG-I**	**MDA5 (IFIH1)**	**LGP2**
**receptors**								
**Downstream Antiviral Genes**		TypeIIFN (IFN-β)	IFNα, IL-1β, PKR, OAS (HPAIV-infected goose)	IL-1β	IL-1, IL-6	IFN-β, IFN-stimulated antiviral genes	IFN-β	IFN-β
**Chicken**	**Genbank accession No.**	NM_001011691; EF137861; DQ780341; JF273967; GU904961.1(emu); XM_002190852.1 (zebra finch).	NM_001011688; GU904977(emu); XM_002194896 (zebra finch).	NM_001037835.1; HM773174.1; HM773175.1; HM773176.1; DQ267901.1; JN544181.1; JN544182.1; JN544183.1; JN544184.1; JN544185.1; JN544186.1; JN544187.1; JN544188.1; JN544189.1;	JQ042910.1; JQ042911.1; JQ042912.1; JQ042913.1; JQ042914.1;	Absent	NM_001193638; JN376086; AB371640; GU570144.	HQ845773
	**Ligand**	dsRNA poly(I:C)	R848 Poly(U)RNA loxoribine	B- and C-type CpG ODNs	CpG ODNs		dsRNA poly(I:C)	dsRNA poly(I:C)
	**Involved in virus infection**	HPAI (H5N1) [37] LPAI (H7N1, H6N2) [45,46] MD [43] IBD [44]	MD [43] IBD [44]	MD [32,33]	Not reported		HPAI [86] LPAI [45]	HPAI [86]
**Duck**	**Genbank accession No.**	JQ910167 (muscovy duck)	DQ888644 (mallard)	Not reported	Not reported	EU363349 (mallard)	GU936632 (mallard)	Not reported
	**Ligand**	dsRNA poly(I:C)	ssPolyU/Lyove loxoribine imiquimod			5′ppp RNA	Unknown	
	**Involved in virus infection**	HPAI (H5N1) [21]	HPAI (H5N1) [56] LPAI (H11N9) [57]			HPAI (H5N1) [75,78-80]	Unknown	
**Goose**	**Genbank accession No.**	Not reported	JQ910168	JQ014619.1	Not reported	HQ829831 (domestic goose)	Not reported	Not reported
	**Ligand**		Not reported	Not reported		5′ppp RNA		
	**Involved in virus infection**		HPAI (H5N1) [26]	Not reported		ND [76]		

^1^ Anas platyrhynchos (mallard); Anser anser (domestic goose); Cairina moschata (Muscovy duck); Dromaius novaehollandiae (emu); Gallus gallus (chicken); Taeniopygia guttata (zebra finch).

^2^ HPAI: Highly pathogenic avian influenza; LPAI: Low pathogenic avian influenza; IBD: Infectious bursal disease; MD: Marek’s disease; ND: Newcastle disease.

The TLR3 ligand poly(I:C) administered as an adjuvant concomitantly with herpesvirus of turkeys (HVT) vaccine in chickens could enhance the protective efficacy against Marek’s disease tumors [40]. A recent study has shown that poly(I:C) has the ability to enhance host immunity against avian influenza viruses (AIV) H4N6 including a significant decrease in virus shedding, upregulation of IFN-α and IL-1β mRNA expression in the spleen, and type I IFN (IFN-α and IFN-β), IL-8, and IL-18 expression in the lungs [41]. These results suggest that TLR3 ligands have the ability to enhance host immunity and could be potential candidates for adjuvants for avian viral vaccines.

Stimulation of chicken monocytes with a CpG-ODN agonist and poly(I:C) either alone or together can produce a stronger T helper cells (Th)1-biased immune response, including upregulation of type I IFN (IFN-α and IFN-β), Th1 cytokines (IFN-γ and IL-12), and Th2 cytokines (IL-4 and IL-10) [42]. It has been suggested that chicken TLR3 and TLR21 agonists may interact to regulate IFN and Th1/2 cytokine expression.

The mRNA expression levels of TLR3 are enhanced in Marek’s disease-infected lungs [43]. However, different strains of infectious bursal disease virus (IBDV) can induce opposite changes of chTLR3 expression levels in the bursa [44]. Highly pathogenic avian influenza virus (HPAIV) H5N1 infection can upregulate chTLR3 and IFN-β expression in the lungs and brain of infected chickens [37], while chTLR3 in the lungs, gut, and bursa is rapidly upregulated by low pathogenic avian influenza virus (LPAIV) H7N1 at the early stages of infection [45]. An in vitro study confirmed that IFN-α and IFN-β are greatly upregulated (up to 10^2^ fold) by poly(I:C) in chicken lung epithelial cells (CLEC213) at 24 h post stimulation, and IFN-α, not IFN-β, is highly expressed (up to 10^7^ fold) in CLEC213 after infection with LPAIV H6N2 [46]. However, the recognition mechanism between AIV and chTLR3 remains to be identified. Moreover, the subsequent activation and cell signal transduction through avian TLR3 remains to be elucidated.

##### 2.3.1.2 Other avian TLR3

The Muscovy duck (Cairina moschata) TLR3 (MdTLR3) (Table 2) shares 87.3% amino acid identity with chTLR3, and 62.0% and 60.2% identity with huTLR3 and mouse TLR3, respectively [21]. It should be noted that the sequence differences between the Muscovy duck and chicken are primarily located in the TIR domain of TLR3, which is among the most conserved TLR sequences.

The expression of MdTLR3 is significantly upregulated in the brain reaching a maximum at 48 h post infection (pi) with HPAIV H5N1, which is similar to chicken TLR3 [21]. In contrast, downregulation of MdTLR3 was observed in the spleen and lung after HPAIV H5N1 infection, while chTLR3 is significantly upregulated in both tissues during infection [21]. Another study indicated that the expression of pigeon (Columbidae) TLR3 [GenBank accession No. AB618533] is upregulated in the brains of HPAIV H5N1 infected pigeons, whereas TLR3 expression is inversely correlated with viral replication in the lungs [47]. In mammals, low expression of TLR3 leads to an unexpected survival advantage, efficient viral replication and decreased lung lesions were confirmed in TLR3-deficient mice after influenza infection [48]. Interestingly, pigeons show more resistance than chickens to HPAIV H5N1 [49]. Collectively, these data suggest that the decrease in TLR3 in the lungs of some birds (such as Muscovy ducks and pigeons) during influenza infection is an important protective host mechanism to prolong survival against influenza, which may explain the different susceptibilities to HPAIV between chickens and waterfowl. However, the detailed molecular mechanism of avian TLR3 action requires further investigation.

#### 2.3.2 Avian TLR7

TLR7, 8, and 9 form an evolutionary cluster and are present in both mammals and teleosteans, and are all localized exclusively in intracellular vesicles such as endosomes and the ER (Figure 1) [10]. TLR7/8 sense viral-derived ssRNA, while TLR9 senses the unmethylated cytosine phosphate guanine (CpG) motifs of viral DNA. Interestingly, only TLR7 has been identified in birds (Table 1 and 2) [17]. TLR8 is a pseudogene in chickens, which is disrupted by several introns [4,17], while it has been proposed that TLR9 has been deleted from avian genomes over evolutionary time [4]. TLR7 orthologs have been found in chicken and zebra finch genomes, but zebra finch TLR7 is duplicated [4].

##### 2.3.2.1 Chicken TLR7

As in mice and humans, chTLR7 is alternatively spliced and expressed as two protein isoforms [17], sharing approximately 63% amino acid identity with huTLR7 [17]. In humans, TLR7 is specifically expressed by plasmacytoid dendritic cells (pDC). ChTLR7 demonstrates a restricted expression pattern, mostly in immune-related tissues [35], as it is highly expressed in the HD11 and DT40 cell lines, but poorly expressed in CEF cells [35]. Another study indicated that chicken erythrocytes constitutively express chTLR2, 3, 4, 5, and 21, but not chTLR7 [50].

In mammals, CL264 (9-benzyl-8 hydroxyadenine), imidazoquinoline, and loxoribine (7-allyl-7,8-dihydro-8-oxo-guanosine) can stimulate TLR7, while resiquimod (R-848) is a TLR7/8 agonist that can induce the activation of NF-κB and MAPK, and the secretion cytokines such as IFN-α, IL-6 and IL-12 via the TLR7/MyD88-dependent signaling pathway [51]. Recently, a study reported that loxoribine induces antiviral gene expression such as type I IFN (IFN-α and IFN-β) and IFN-γ in primary chicken splenocytes, and can inhibit influenza A replication in vitro and in ovo in a dose-dependent manner [52]. However, R848 and poly(U) cannot stimulate chicken splenocytes to upregulate the expression of type I IFN, but it can cause increased expression of IL-1β, IL-6 and IL-8 [17]. Moreover, IL-1β mRNA levels in HD11 cells were significantly increased after R848 or loxoribine stimulation for 6 h [17]. In contrast, another study indicated that IL-1β, IL-6 and IL-8 mRNA levels were significantly decreased in heterophils after stimulation with loxoribine for 1 h irrespective of concentration (20 to 200 μg/mL) [53]. Subsequently, it was demonstrated that chicken heterophils from different commercial poultry suppliers respond differently to TLR7 agonists (loxoribine) in relation to IFN-α, proinflammatory cytokines (IL-1β and IL-6) and chemokine expression (CXCLi2, CXCLi1 and CCLi4) [54]. Intriguing differences in the functional responses to TLR7 agonists have been observed either in different types of chicken cells or in heterophils from different parent lines. However, the underlying causes of these different responses are not yet understood.

The expression of chTLR7 is upregulated in the lungs post MDV infection [43]. Similar to chTLR3, chTLR7 expression varies in response to different strains of IBDV infected chickens [44]. These data indicate that chTLR7 is involved in the interaction between the host and RNA viruses.

##### 2.3.2.2 Duck TLR7

DuTLR7 (White Pekin Duck) shares approximately 85% amino acid identity with chTLR7, differing primarily in the ligand-binding LRR domains [23]. DuTLR7 transcripts are most abundantly expressed in lymphoid tissues such as the spleen and bursa, as well as the lung [23]. It is very interesting to note that the high expression of duTLR7 in respiratory and lymphoid tissues is distinct from that of chickens, yet a similar expression pattern is observed in humans [23,55]. Moreover, proinflammatory cytokines (IL-1β and IL-6) and IFN-α are upregulated in duck splenocytes after stimulation with TLR7 agonists (loxoribine and imiquimod), which appear to be critical mediators of antiviral defenses (Table 2) [23]. Tissue tropism and immune function of duTLR7 is different from that of chTLR7, which may partly explain the different susceptibility between chickens and ducks to the same pathogen.

During HPAIV H5N1 infection, type I IFN (IFN-α and IFN-β), type II interferon (IFN-γ) and TLR7 were slightly upregulated in DEF, whereas expression was only gradually induced in CEF [56]. Another study demonstrated that both duck and chicken TLR7 is only transiently expressed in peripheral blood mononuclear cells (PBMC) at the early stages of LPAIV H11N9 infection, followed by a decline as the infection progresses [57]. These results demonstrate that there are some differences in the changes in TLR7 expression in ducks and chickens. Regulation of chTLR7 expression during HPAIV or LPAIV infection was also different. It has been suggested that TLR7 is an important element in the avian immune response against influenza virus and that TLR7 ligands show considerable potential as antivirals in the chicken.

##### 2.3.2.3 Goose TLR7

GoTLR7 (Qingyuan Goose) shares only 89% and 68% sequence identity with chTLR7 and huTLR7 [26]. We have shown that goTLR7 (Sichuan White Goose) shares 93% and 83% amino acid identity with duTLR7 [GenBank accession No. ABK51522.1] and chTLR7 [GenBank accession No. CAG15146.1], respectively (data not shown). As in ducks, goTLR7 is predominantly expressed in lymphoid tissues (such as the bursa), while expression is observed in the intestines and lungs as well (data not shown). The expression of goTLR7, MyD88 and other antiviral molecules were significantly upregulated in the lungs during the early phase of HPAIV H5N1 infection [26]. HPAIV H5N1 can activate goTLR7 via the MyD88-dependent pathway, and goTLR7 is involved in the antiviral innate immune response against virus. However, the interaction between HPAIV and goTLR7 requires further study. The immunological characteristics of goTLR7 remain to be elucidated as well.

#### 2.3.3 Mammalian TLR9 and avian TLR21

CpG-ODN are common in bacteria and viruses but are rare in mammalian cells, and are therefore a major ligand of mammalian TLR9, as TLR9-deficient mice fail to respond to CpG-ODN [58]. In mammals, high TLR9 expression in pDC serves as a sensor for DNA virus infection (such as HSV1 and HSV2) by exerting an effective antiviral immune response by producing type I IFN [59,60]. It is thought that the CpG-rich genomes of some DNA viruses are recognized by TLR9. Interestingly, the chicken genome does not contain TLR9, however the administration of CpG-ODN in ovo can enhance the expression of IFN-γ, IL-1β, IL-6, and IL-8, as well as limit the propagation of infectious bronchitis virus (IBV) in embryonic tissues [61]. Investigation of the immunoadjuvant effects of CpG-ODN on the Newcastle disease (ND) vaccine indicated that CpG-ODN can induce PBMC proliferation and higher ND vaccine-specific antibody titers [62]. Another study indicated that CpG-ODN could enhance the humoral immune response (HI titres and serum IgG titres) and Th1 immunity (IFN-γ) when administered with the AIV vaccine as the adjuvant [63]. These results suggest that CpG-ODN administered as an adjuvant with poultry vaccines can greatly improve their effectiveness. These findings lead to a search for other receptors or TLR in birds (such as TLR7 or the novel TLR15 or TLR21) that can recognize mammalian TLR9 ligands (such as CpG-ODNs) to trigger downstream cell signaling.

It was later confirmed that chTLR21, not chTLR7 or chTLR15, has an immune function similar to mammalian TLR9 and an innate CpG DNA receptor that can recognize bacterial genomic DNA [19,64]. TLR21 is absent in humans, but has been identified in chickens and turkeys, and has homologs in amphibians (*Xenopustropicalis* (61% identity)) and fish (Takifugu rubripes (57% identity)), and is similar to murine TLR13 (47%) [65]. Like huTLR9, chTLR21 is located in the same intracellular compartment (i.e. the ER) in transfected cells as confirmed by confocal microscopy [19,64]. It has also been demonstrated that chicken intestinal T-cell subsets (CD4+ and CD8+ T cells) express chTLR21 [66]. The expression of chTLR21 was the highest in the bursa of Fabricius and the spleen, and was also present in non-lymphoid tissues (skin, small intestine, lung, kidneys, brain and liver) of adult chickens [19], while levels of turkey TLR21 were significantly higher in the liver than other tissues such as muscle, intestine, bursa and spleen [30]. Due to selection pressures during vertebrate evolutionary history, different classes of TLR were formed that recognize the same type of ligands and show analogous localization (such as chicken TLR21 and mammalian TLR9). Functionally, avian TLR21 and mammalian TLR9 are both involved in CpG-ODN sensing and the immune response to viral infections.

## 3. Rig-I-like receptors for cytosolic sensing

The RLR family consists of three members: RIG-I, melanoma differentiation-associated gene 5 (MDA5) and laboratory of genetics and physiology 2 (LGP2) [67]. RIG-I and MDA5 contain two N-terminal caspase activation and recruitment domains (CARD), a DEX(D/H) box RNA helicase domain, a C-terminal RNA-binding domain and a repressor domain, whereas LGP2 lacks a CARD [68]. MDA5 and LGP2 are well conserved among vertebrates, while RIG-I emerged earlier than MDA5 and LGP2 [69].

### 3.1 RIG-I

RIG-I belongs to the IFN-stimulated gene (ISG) family and is expressed ubiquitously in the cytoplasm (Figure 1) [67]. RIG-I detects viral nucleic acids in the cytosol and activates a downstream signaling cascade, initiating antiviral responses by producing type I and type III IFN (Figure 1) [67]. It is specific to RNA viruses and does not bind viral dsDNA except for the Epstein-Barr virus [70]. RIG-I recognizes dsRNA structures and short, blunt-ended 5′-triphosphate (5′ppp) dsRNA generated by viral RNA polymerases, but viral RNA that contain a 5′-monophosphate are not recognized by RIG-I [68,71]. Several viruses remove their own 5′ppp groups to avoid detection by RIG-I [68]. All eukaryotic cellular mRNA are blocked at their 5′-ends with a cap structure to prevent sensing by RIG-I. ATRA, LPS, type I IFN (IFN-α and IFN-β) and type II IFN (IFN-γ) can upregulate RIG-I expression in a variety of human cell types [72]. RIG-I expression is subject to positive feedback regulation by IFN, which means that RIG-I is an IFN-inducible viral sensor and is critical for amplifying antiviral responses [73,74]. The stickleback genome appears to lack RIG-I, and incomplete sequence data means that the RIG-I gene could not be identified in Fugu, medaka and chicken [69,75]. However, RIG-I has been identified in both ducks and geese [75,76]. The structure of avian RIG-I is consistent with its mammalian counterpart [69].

#### 3.1.1 Duck RIG-I

Duck (*Anas platyrhynchos*), zebra finch, and goose RIG-I are 933 [75], 927, and 934 amino acids in length, respectively [76]. Duck RIG-I shares 78% and 53% amino acid identity with zebra finch RIG-I and human RIG-I, respectively [75].

A study demonstrated that RIG-I expression is dramatically upregulated in the lungs of HPAIV H5N1-infected ducks, but not in LPAIV H5N2-infected ducks [75]. Duck RIG-I-transfected DF-1 cells can recognize the RIG-I ligand and induce the expression of antiviral genes including chicken IFN-β, MX1, PKR, IFIT5, and OASL, while HPAIV titers are significantly reduced [75,77]. Duck and wild waterfowl are the natural reservoirs of influenza viruses, that usually cause asymptomatic infections in ducks, while causing lethal infections in chickens. Despite the lack of RIG-I, chicken cells can produce IFN-α by another pathway. However, the expression of IFN-β upon influenza infection is mainly dependent on RIG-I [78], and the protective role of IFN-β during influenza infection cannot be compensated for by IFN-α [79]. This may contribute to the higher resistance of ducks than chickens to HPAIV infections.

#### 3.1.2 Goose RIG-I

Goose RIG-I exhibits 93.8%, 78.1% and 50.8% amino acid identity with duck, zebra finch and human RIG-I, respectively [76,80]. Goose RIG-I is highly expressed in the lungs, liver, brain, and the bursa of Fabricius, but poorly expressed in the thymus and intestines of 1 week old goslings [80].

Newcastle disease virus (NDV) is a contagious and fatal viral disease affecting most species of birds including chickens, while ducks and sand geese are naturally resistant to it. Similar to the inverse correlation between the susceptibility to HPAIV and the basal expression of duck RIG-I, a strong inverse correlation between NDV susceptibility and goose RIG-I expression was observed [75,76]. Goose RIG-I-transfected 293 T/17 cells and DF-1 cells can both respond to NDV by upregulating the activity of the IFN-β promoter and expression levels of IRF-3 and IFIT-1, while decreasing the viral titer [76]. Further, goose RIG-I mRNA levels are increased upon NDV infection in vivo [76]. Another study also indicated a direct correlation between cellular resistance to NDV infection and RIG-I expression in vitro [81]. It is suggested that there is an inverse correlation between susceptibility to NDV infection and the endogenous expression of RIG-I in birds. These findings offer further insights into the mechanism by which waterfowl are natural resistant to NDV and HP AIV infections.

### 3.2 MDA5

In humans, MDA5 and RIG-I are members of an evolutionary conserved family. MDA5 and RIG-I are key cytosolic PRR that detect the nucleic acids of invading viruses and lead to the activation of the interferon system (Figure 1) [8,67,68]. MDA5 can be activated by dsRNA, including the synthetic dsRNA analogue poly(I:C) [82]. Long poly(I:C) sequences (>1 kbp) are selectively recognized by MDA5, whereas relatively short poly(I:C) sequences generated by enzyme digestion (<1 kbp) are recognized by RIG-I (Figure 1) [82]. Therefore, viral RNA in infected cells is selectively recognized by RIG-I and MDA5, depending on its length. The activation of RIG-I and MDA5 initiate signal transduction, resulting in the secretion of type I IFN and various cytokines (Figure 1).

#### 3.2.1 Chicken MDA5

Chickens, unlike ducks, lack RIG-I, however MDA5 transcripts are present and functionally compensate for the absence of RIG-I (Tables 1 and 2) [69,75]. Human and chicken MDA5 share 60% amino acid identity, and the C-terminal region of MDA5 shows a relatively high degree of conservation (about 70% identity) [83]. Chicken MDA5 (chMDA5) is ubiquitously expressed in several tissues, with the highest expression in the intestines [84].

In mammals, RIG-I is the main cytosolic PRR for the detection of influenza A virus [68]. HPAIV H5N1 tempers the human innate immune response by downregulating the expression of endogenous RIG-I, which is mediated through inhibition of IFN-β production [85]. In chickens, MDA5 is the primary cytosolic influenza A virus sensor in chicken cells including DF-1 fibroblasts and HD11 cells [86]. Influenza A virus dsRNA is recognized by chMDA5 resulting in type I IFN induction, which involves chicken LGP2, CARDIF and IRF3 [86]. The overexpression of chMDA5 in DF-1 cells induces the activation of the chicken IFN-β promoter in the absence of dsRNA agonist stimulation in a species-specific manner and can be inhibited by the V proteins of human paramyxovirus (PIV5) and NDV [87]. RNAi-mediated knockdown experiments further suggest that chMDA5 plays an important role in the IFN response of chicken cells to dsRNA, but the proliferation of influenza A viruses is unaffected [86]. Moreover, another study indicated that the N-terminal 483 amino acids of chMDA5 can trigger IFN-β responses in chicken cells, and can therefore work as an adjuvant and be co-administered with DNA vaccines against H5N1 HPAIV to enhance vaccine efficacy [88]. Furthermore, it was shown that chickens and ducks show differential MDA5 expression in the presence of LPAIV [45]. Interestingly, MDA5 and RIG-I can distinguish different RNA viruses in mammals [89], however the immunological characteristics of duck MDA5 are not known.

### 3.3 Chicken LGP2

LGP2, the third member of the RLR family, lacks a CARD and has a greater affinity for dsRNA than RIG-I, but retains the RNA-binding domain, thus it is a negative regulator of RLR-mediated signaling [90]. The putative amino acids of chicken LGP2 (chLGP2) show 53% and 52% identity with human and mouse LGP2, respectively [87]. The siRNA-mediated knockdown of chLGP2 in chicken cells reduced HPAIV H5N1-induced type I IFN secretion, while the overexpression of chLGP2 reduced the activation of the chicken IFN-β promoter [87]. This indicates that chLGP2 functions are required for chicken RLR signaling, similar to mammalian LGP2 [90]. It is worth noting that many immunological characteristics of chicken LGP2 are not yet understood, and whether or not MDA5 and LGP2 are present in other avian species remains to be discovered.

The interactions between influenza or other RNA viruses and RIG-I, MDA5 or LGP2 are largely unknown. After recognition, the subsequent cell signal transduction and the release of effect factors also need to be studied in detail.

## 4. Nod-like receptors

NLR primarily recognize microbial molecules of bacterial origin that are used in antibacterial defenses [91]. However, in mammals, NLR sense several different RNA or DNA viruses, and positively or negatively regulate the innate antiviral response [92]. The human NLR family is composed of 23 members, whereas there are at least 34 members in the mouse NLR family [92], several of which respond to the various PAMP, non-PAMP particles and cellular stresses which trigger proinflammatory responses, including the secretion of IL-1β and IL-18 [92,93]. NLR are composed of three major domains: C-terminal leucine-rich repeats (LRR) recognizing PAMP ligands, a central nucleotide-binding oligomerization (NOD) domain that mediates self-oligomerization during activation, and a variable N-terminal protein-protein interaction domain involved in signal transduction [91]. Based on the N-terminal effector domains including the caspase recruitment domain (CARD), pyrin domain (PYD), acidic transactivating domain, or baculovirus inhibitor repeat (BIR), the NLR can be classified into three subfamilies [94].

### 4.1 NLRC5

NLRC5, a member of the NLR family, is expressed in most cell types as an intracellular receptor, but the role of NLRC5 in inflammatory immune responses is controversial in humans [95]. NLRC5 is a positive IFN mediator in response to viral infection [96], while it attenuates the antiviral response to VSV [97]. In chickens, the inhibitory effects of NLRC5 on inflammatory pathways are well understood [98,99]. The expression of chicken NLRC5 is greatly increased in LPS-treated HD11 cells, but not in poly(I:C)-treated HD11 cells. The knockdown of NLRC5 expression downregulates the expression of type I IFN (IFN-α and IFN-β), but not IL-6 and MHC class I in chicken HD11 cells [98]. Also, NLRC5 is induced in response to *Salmonella* endotoxin in HD11 cells [99]. These findings may indicate that chicken NLRC5 is involved in the host antimicrobial immune response and may contribute to the design of efficacious poultry vaccines. Many members of the avian NLR family are still not identified. However, the role for avian NLR in viral immunity is largely unknown.

## 5. Conclusions

In the past decade, there has been much progress in understanding the roles of pattern recognition receptors in viral recognition and host defenses. Similar to mammals, three classes of receptors including TLR, RLR and NLR have been identified in birds. Some of these receptors are involved in host antiviral immune responses through the regulation of interferons and proinflammatory cytokines. The rapid mobilization of type I and type III IFN is critical for host immunity against viral infection.

However some differences between avian and mammalian TLR include the pseudogene TLR8, the absence of TLR9, and the presence of TLR1La, TLR1Lb, TLR15, and TLR21 in birds. Avian TLR3 and TLR7 are involved in RNA virus recognition including AIV, MDV and IBDV. It seems that avian TLR15 and TLR21, like mammalian TLR4 or TLR9, are potential sensors recognizing invading microbes including viruses and bacteria. Moreover, some viral sensing receptors are slightly different between fowl and waterfowl. Chickens, unlike ducks, geese and finches, lack RIG-I, however MDA5 transcripts are present and functionally compensate for the absence of RIG-I. The expression of type I IFN upon HPAIV infection in chickens and ducks is dependent upon MDA5 and RIG-I, respectively. The expression of IFN-β upon influenza infection is mainly regulated by RIG-I leading to expression of downstream IFN-stimulated antiviral genes. This may explain the differing susceptibilities to HPAIV H5N1 in the chicken and other birds. NLRC5, the only member of the NLR family that has been identified in birds, is reported to be involved in the host antiviral immune response.

Although some viral sensing PRR and their cognate viral PAMP have been identified in birds, there is still a long way to go toward a complete understanding of the viral recognition mechanism and antiviral response of the host, especially in waterfowl. The ontogeny and the cellular localization of avian PRR have yet to be confirmed. It is unknown yet whether other PRR that are absent in mammals are present in birds. Moreover, many signaling molecules and downstream signaling pathways activated by various PRR and the cross talk between them also need to be elucidated.

Birds, especially migratory birds, are an important reservoir of viruses causing human infections and are a valuable food source. Further understanding of the antiviral function of avian PRR could lead to the development of novel vaccines and strategies for controlling infectious diseases.

## 6. Competing interests

The authors declare that they have no competing interests.

## 7. Authors’ contributions

SC developed the structural design of the review and drafted the manuscript. AC and MW were involved in revising the manuscript critically for important intellectual content. All authors read and approved the final manuscript.
